# Inhibition of HIV Replication by Apolipoprotein A-I Binding Protein Targeting the Lipid Rafts

**DOI:** 10.1128/mBio.02956-19

**Published:** 2020-01-21

**Authors:** Larisa Dubrovsky, Adam Ward, Soo-Ho Choi, Tatiana Pushkarsky, Beda Brichacek, Christophe Vanpouille, Alexei A. Adzhubei, Nigora Mukhamedova, Dmitry Sviridov, Leonid Margolis, Richard B. Jones, Yury I. Miller, Michael Bukrinsky

**Affiliations:** a The George Washington University School of Medicine and Health Sciences, Washington, DC, USA; b University of California San Diego, La Jolla, California, USA; c Eunice Shriver National Institute of Child Health and Human Development, National Institutes of Health, Bethesda, Maryland, USA; d Engelhardt Institute of Molecular Biology, Russian Academy of Sciences, Moscow, Russia; e Baker Heart and Diabetes Research Institute, Melbourne, Victoria, Australia; University of Pittsburgh School of Medicine

**Keywords:** HIV, AIBP, Nef, extracellular vesicles, exosomes, lipid rafts, fusion, HLA

## Abstract

Apolipoprotein A-I binding protein (AIBP) is a protein involved in regulation of lipid rafts and cholesterol efflux. AIBP has been suggested to function as a protective factor under several sets of pathological conditions associated with increased abundance of lipid rafts, such as atherosclerosis and acute lung injury. Here, we show that exogenously added AIBP reduced the abundance of lipid rafts and inhibited HIV replication *in vitro* as well as in HIV-infected humanized mice, whereas knockdown of endogenous AIBP increased HIV replication. Endogenous AIBP was much more abundant in activated T cells than in monocyte-derived macrophages (MDMs), and exogenous AIBP was much less effective in T cells than in MDMs. AIBP inhibited virus-cell fusion, specifically targeting cells with lipid rafts mobilized by cell activation or Nef-containing exosomes. MDM-HIV fusion was sensitive to AIBP only in the presence of Nef provided by the virus or exosomes. Peripheral blood mononuclear cells from donors with the HLA-B*35 genotype, associated with rapid progression of HIV disease, bound less AIBP than cells from donors with other HLA genotypes and were not protected by AIBP from rapid HIV-1 replication. These results provide the first evidence for the role of Nef exosomes in regulating HIV-cell fusion by modifying lipid rafts and suggest that AIBP is an innate factor that restricts HIV replication by targeting lipid rafts.

## Abstract (precis)

Apolipoprotein A-I binding protein (AIBP) is a recently identified innate anti-inflammatory factor. Here, we show that AIBP inhibited HIV replication by targeting lipid rafts and reducing virus-cell fusion. Importantly, AIBP selectively reduced levels of rafts on cells stimulated by an inflammatory stimulus or treated with extracellular vesicles containing HIV-1 protein Nef without affecting rafts on nonactivated cells. Accordingly, fusion of monocyte-derived macrophages with HIV was sensitive to AIBP only in the presence of Nef. Silencing of endogenous AIBP significantly upregulated HIV-1 replication. Interestingly, HIV-1 replication in cells from donors with the HLA-B*35 genotype, associated with rapid progression of HIV disease, was not inhibited by AIBP. These results suggest that AIBP is an innate anti-HIV factor that targets virus-cell fusion.

## INTRODUCTION

Apolipoprotein A-I binding protein (AIBP) was discovered in a screen of proteins that physically associate with apolipoprotein A-I (1). Although intracellular functions of AIBP have been proposed previously (2, 3), it is well established that secreted AIBP regulates cholesterol trafficking and lipid rafts in the plasma membrane in vertebrate animals (4–9). The mechanisms of AIBP secretion are not completely understood. The protein was not found in plasma of healthy subjects but was detected in plasma of sepsis patients (1). ApoA-I and high-density lipoproteins (HDL) induced AIBP secretion from cells of the kidney proximal tubules (1), and lipopolysaccharide (LPS) induced AIBP secretion from murine alveolar macrophages (8). Experiments performed *in vitro* and in animal models suggest that AIBP enhances ApoA-I-mediated cholesterol efflux specifically from cells (endothelial cells, macrophages, and microglia) challenged by proinflammatory agents (“activated” cells) while sparing nonactivated cells (5–9). Thus, AIBP appears to selectively target lipid rafts on activated cells, normalizing their abundance and function activated by inflammatory stimuli (7). In this work, we tested the hypothesis that AIBP may modulate HIV infection via regulation of lipid rafts in host cells.

Host cell lipid rafts are critically important for the biology of HIV. Both HIV-1 assembly and budding occur at lipid rafts of infected cells, and infection of target cells also involves lipid rafts (10–12). Given the key role of lipid rafts in HIV replication, it is not surprising that HIV has evolved to acquire mechanisms regulating the abundance of these membrane domains, mainly via the effects of HIV protein Nef. Nef has been shown to inhibit the activity of ABCA1 cholesterol transporter and to suppress cellular cholesterol efflux mediated by this factor (13), to stimulate cholesterol biosynthesis (14, 15), and to deliver cholesterol to lipid rafts, increasing their abundance (14, 16, 17). Importantly, Nef can inhibit ABCA1 and cholesterol efflux not only in HIV-infected cells but also in bystander cells, which are naturally resistant to HIV infection (18, 19). This systemic effect of Nef is due to release of this protein from infected cells into the blood in extracellular vesicles, which then deliver this protein throughout the body (20, 21). It is not surprising that HIV replication can be inhibited by pharmacologic agents that reduce the abundance of lipid rafts. For example, cyclodextrin severely impairs HIV infectivity (22) and inhibits HIV production (23). Topical application of 2-hydroxypropyl-β-cyclodextrin was previously shown to block vaginal transmission of cell-associated HIV-1 in a humanized mouse (hu-mouse) model (24). The anti-HIV activity of statins involves downmodulation of lipid raft formation (25, 26). Our previous studies demonstrated that stimulation of expression of cholesterol transporter ABCA1 by agonists of liver X receptor (LXR), which leads to reduction of lipid raft abundance, potently inhibits HIV-1 replication (27, 28). However, the use of cyclodextrins, statins, or LXR agonists does not afford selectivity for cells infected with HIV or exposed to pathogenic factors released from infected cells.

Given the dependence of HIV on lipid rafts and the ability of AIBP to reduce the abundance of lipid rafts, in this study we investigated whether such activity of AIBP translates into inhibition of HIV replication. We found that AIBP inhibits HIV replication both *in vitro* and *in vivo* and reverses the proinfectious effects of Nef-containing extracellular vesicles. New therapeutic approaches aimed at inhibition of HIV infection and HIV-associated comorbidities via stimulation of AIBP production can be envisioned.

## RESULTS

### AIBP inhibits HIV replication *in vitro*.

**FIG 1:**
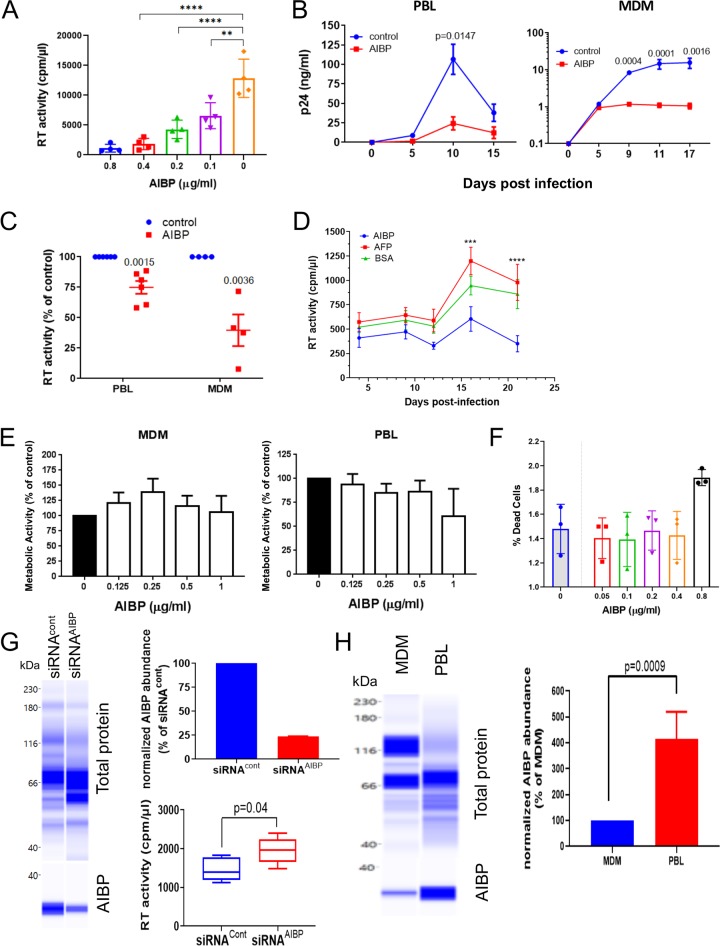
AIBP inhibits HIV-1 replication. (A) Quadruplicate wells of monocyte-derived macrophages (MDMs) were infected with HIV-1_ADA_, and virus production was measured on day 12 postinfection by analysis of RT activity in culture supernatant. Bars show means ± standard deviations (SD). Statistical analysis was done by Dunnett’s multiple-comparison test. **, *P* = 0.0013; ****, *P* < 0.0001. (B) PHA-activated PBLs or MDMs from one representative donor were infected in triplicate wells with HIV-1_LAI_ or HIV-1_ADA_ strains, respectively, cultured in the presence or absence of recombinant AIBP (0.2 μg/ml), and HIV replication was followed by measuring p24 levels in culture supernatants. Holm-Sidak-adjusted *P* values from multiple-comparison test are shown. (C) The experiment was performed as described for panel B with PHA-activated PBLs from 6 donors and MDMs from 4 donors. Virus replication was followed by analysis of RT activity. Results are presented for each donor at the peak of infection as percent RT activity in AIBP-negative (control) culture. Holm-Sidak-adjusted *P* values are shown. (D) PHA-activated PBLs were infected with T/F strain pCH185.c/K3016 and cultured in the presence or absence of 0.2 μg/ml recombinant AIBP (recombinant AFP was used as a control). Virus replication was followed by analysis of RT activity. Results show means ± SD (*n* = 4). Holm-Sidak-adjusted *P* value is shown. (E) PHA-activated PBLs and MDMs were exposed to the indicated concentrations of recombinant AIBP for 3 days, the cytotoxic effect of AIBP was measured by MTT assay, and the results are presented as percent metabolic activity of AIBP-negative (control) cultures. Bars show means ± SD (*n* = 4). (F) PHA-activated PBLs were treated with AIBP as described for panel C, and percentages of live cells were measured by flow cytometry using a LIVE/DEAD Fixable Aqua kit (Invitrogen). Bars show means ± SD (*n* = 3). (G) PHA-activated PBLs were treated with AIBP-targeting or control Accell siRNA for 72 h. AIBP abundance was measured by ProteinSimple Western blotting (left panel) and normalized against total protein, and data are presented relative to the results seen with cells treated with siRNA^Cont^ (right top panel). Cells were infected in 5 wells with HIV-1_LAI_ and cultured for 3 days, and HIV production was measured by analysis of RT activity in culture supernatant (right bottom panel). *P* values were calculated by unpaired *t* test (*n* = 5). (H) PBLs and MDMs from the same donor were analyzed in triplicate for AIBP by ProteinSimple Western blotting (left panel). Relative abundances of AIBP normalized against total protein are shown in the right panel. *P* values were calculated by unpaired *t* test (*n* = 3).

In previous studies, an AIBP concentration of 0.2 μg/ml was found to be an effective dose for stimulation of cholesterol efflux from myeloid cells (5, 6, 8, 9). Note that AIBP activity depends on the presence of ApoA-I; thus, all experiments measuring functional activity of AIBP were done in the presence of 10% human serum as a source of ApoA-I. To test the effect of AIBP on HIV replication, we first performed titrations to determine the effect of baculovirus-expressed recombinant AIBP on infection of monocyte-derived macrophages (MDMs) by HIV-1 ADA. The results presented in Fig. 1A demonstrated that at a 0.2 μg/ml concentration, AIBP significantly reduced the amount of HIV-1 in the culture medium at day 12 postinfection (p.i.). To be consistent with previous studies, we chose this concentration for subsequent experiments.

Analysis of HIV replication kinetic in peripheral blood lymphocyte (PBL) and MDM cultures infected with HIV-1 strains X4 (LAI) and R5 (ADA), respectively, demonstrated significant suppression by AIBP of HIV-1 replication in both PBLs and MDMs (Fig. 1B). As a control in the initial experiments, we used baculovirus-expressed recombinant human alpha-fetoprotein (AFP). This protein produced no effect on HIV replication in PBLs (Fig. 1D; see also Fig. S2 in the supplemental material), so bovine serum albumin (BSA) was used as a negative control in most subsequent experiments. The mean levels of inhibition of HIV replication by AIBP relative to cultures with no added AIBP calculated with cells from several different donors were over 50% for MDM cultures and about 25% for PBL cultures (Fig. 1C).

10.1128/mBio.02956-19.2 FIG S2 AIBP inhibits HIV-1 replication. PHA-activated PBMCs were infected in quadruplicate wells with HIV-1 LAI and cultured for 5 days in the presence of 0.2 μg/ml of BSA, baculovirus-expressed alpha-fetoprotein (AFP), or baculovirus-expressed AIBP. Virus production was measured by analysis of RT activity in culture supernatant. Results are presented as means ± SD. *P* values were calculated by ordinary one-way ANOVA with Tukey’s correction for multiple comparisons. Download FIG S2, PDF file, 0.6 MB. Copyright © 2020 Dubrovsky et al. 2020 Dubrovsky et al. This content is distributed under the terms of the Creative Commons Attribution 4.0 International license.

Experiments using laboratory-adapted HIV strains, such as R5-ADA and X4-LAI used as described above, are less reliably predictive of *in vivo* outcomes than those using strains mediating transmission between HIV-positive individuals, also known as transmitted/founder (T/F) viruses, which consistently display CCR5 coreceptor tropism (29, 30). We thus tested the effect of AIBP on replication of the primary T/F virus, the CCR5-tropic strain pCH185.c/K3016 (31). This virus did not replicate in MDMs, consistent with a previous report (31) and with *ex vivo* data showing that the primary targets of transmitted HIV-1 are CD4^+^ T cells and not macrophages (32). In PBLs, AIBP noticeably suppressed replication of pCH185.c/K3016 (Fig. 1D). No toxicity of AIBP was revealed by 3-(4,5-dimethyl-2-thiazolyl)-2,5-diphenyl-2H-tetrazolium bromide (MTT) assay (Fig. 1E), and LIVE/DEAD flow cytometry assay confirmed exclusion of necrotic and apoptotic cells (Fig. 1F). Finally, to determine whether endogenously expressed AIBP exerts anti-HIV activity, we treated PBLs with AIBP-targeting or control small interfering RNA (siRNA) prior to infection with HIV-1. This treatment reduced the abundance of AIBP by over 70% in cells treated with AIBP-specific siRNA relative to control (scrambled) siRNA (Fig. 1G, left and top right panels). HIV replication was significantly increased in cells with knocked down AIBP (Fig. 1G, bottom right panel). Of note, AIBP expression in phytohemagglutinin (PHA)-activated PBLs was substantially higher than in MDMs (Fig. 1H), explaining the difference between these cell types in the magnitude of anti-HIV activity of exogenously added AIBP. Overall, although the observed level of inhibition was relatively modest, it provided the first evidence that AIBP can exert anti-HIV activity. The robustness of this observation was supported by results from multiple HIV-1 strains susceptible to the AIBP-mediated inhibitory activity, which was reproduced with cells from multiple donors.

### AIBP targets lipid rafts.

**FIG 2:**
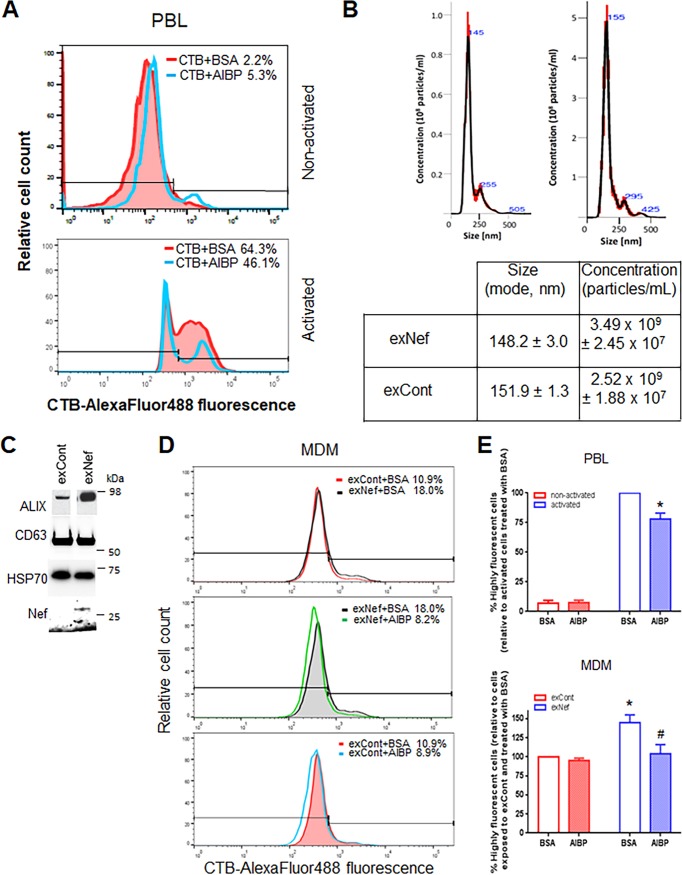
AIBP regulates abundance of lipid rafts. (A) PBLs from a representative donor were stimulated with PHA or left unstimulated, stained with fluorescently labeled cholera toxin subunit B, and analyzed by flow cytometry. (B) Representative analysis of vesicle size and concentration in exosome samples from supernatants of HEK293 T cells transfected with Nef (exNef) or empty vector (exCont) by Nanosight (top panels). Means ± standard errors of the means (SEM) of vesicle size (in nanometers) and vesicle concentration (in particles per milliliter) are shown in the bottom panel. (C) Vesicles were analyzed by Western blotting for the exosomal marker Alix, tetraspanin CD63, cytosolic marker HSP70, and Nef. (D) MDMs from a representative donor were treated with exNef or exCont in the presence of AIBP or BSA, and lipid rafts were analyzed as described for panel A. (E) Lipid rafts were analyzed as described for panels A and D. Results are presented for experiments performed with cells from 4 different donors. (Left panel) *, *P* = 0.0088 (unpaired *t* test performed with Holm-Sidak adjustment, relative to activated PBLs treated with BSA). (Right panel) *, *P* = 0.0038 (relative to cells treated with exCont and BSA); #, *P* = 0.0129 (relative to MDMs treated with exNef and BSA; ordinary one-way ANOVA with Tukey’s adjustment for multiple comparisons).

Given that AIBP reduces the abundance of lipid rafts (5), we hypothesized that the mechanism of HIV inhibition by AIBP might involve suppression of virus-cell fusion, which relies on rafts (33). Previous studies demonstrated that AIBP reduces abundances of lipid rafts on activated but not on nonactivated macrophages and endothelial and glial cells (5, 7). Nothing has been reported about the effects of AIBP on T cells. Using fluorescently labeled cholera toxin subunit B (CTB), which specifically binds to the raft-associated ganglioside GM1 (34) and is commonly used to assess lipid raft abundance (35–37), we evaluated the effect of AIBP on lipid rafts of PBLs activated or not activated with PHA. In nonactivated PBLs, the percentage of cells with high-intensity signal was very small, suggesting a low abundance of lipid rafts (consistent with previously reported findings [38, 39]), and AIBP did not decrease the raft abundance (in fact, in this donor, AIBP increased the abundance of the rafts) (Fig. 2A; see also Fig. S3A and B). In PHA-activated cells, in contrast, the abundance of rafts was high and was reduced by AIBP (Fig. 2A).

10.1128/mBio.02956-19.3 FIG S3 Gating strategy for Fig. 2. (A) Gating for unactivated PBL (Fig. 2A). (B) Gating for activated PBL (Fig. 2A). (C) Gating for MDM (Fig. 2C). Download FIG S3, PDF file, 0.6 MB. Copyright © 2020 Dubrovsky et al. 2020 Dubrovsky et al. This content is distributed under the terms of the Creative Commons Attribution 4.0 International license.

AIBP has been reported to reduce the abundance of rafts in macrophages on LPS-stimulated cells (7). However, LPS inhibits HIV infection of macrophages by downregulating CCR5 and inducing postentry degradation of viral RNA; therefore, increased abundance of lipid rafts in LPS-treated macrophages does not translate into increased HIV infection (40, 41). Another agent upregulating lipid rafts on macrophages is the HIV protein Nef (17). Our recent study (42) demonstrated that the same effect on lipid rafts is produced by Nef-containing exosomes (exNef). We collected exNef from supernatants of HEK293T cells transfected with Nef_NL4-3_-expressing vector. Control exosomes (exCont) were collected from HEK293T cells transfected with empty vector. Of note, no difference was found between the effects on ABCA1 and lipid raft abundance induced by exosomes produced by cells transfected with empty vector and the effects induced by vector expressing green fluorescent protein (GFP) (used as a control in the previous study) (42). We next analyzed the exosomes using Nanosight (Fig. 2B). The majority of exosomes had the size of 150 nm characteristic for these vesicles (43). Consistent with this classification, the vesicles were positive for exosomal markers ALIX and tetraspanin CD63 and also carried cytoplasmic protein Hsp70 (Fig. 2C), thus fulfilling the requirements of the International Society for Extracellular Vesicles (ISEV) for exosome purity (44). Treatment of macrophages with exNef increased the proportion of highly fluorescent CTB-stained cells from 10.9% to 18.0% (Fig. 2D, top panel; see also Fig. S3C). Remarkably, added recombinant AIBP reduced the abundances of lipid rafts on exNef-treated macrophages to the levels observed in nonactivated cells (Fig. 2D, middle panel). Statistical analysis performed with cells from 4 different donors confirmed that AIBP did not significantly change the abundance of lipid rafts on nonactivated PBLs but significantly (*P* = 0.0088) reduced the abundance of rafts on PHA-activated PBLs (Fig. 2E, left panel) and on exNef-treated MDMs (*P* = 0.0129) (Fig. 2E, right panel). Of note, exNef significantly (*P* = 0.0038) increased the abundance of lipid rafts on MDMs (Fig. 2D, top panel, and 2E, right panel). This result is consistent with our recent report (42), where we also demonstrated that the effect on lipid rafts of exNef was identical to the effect of exosomes produced by cells infected with Nef-expressing HIV-1. The effect of AIBP on lipid rafts in macrophages that were exposed to control exosomes was not statistically significant (Fig. 2E, right panel). Together, these results are consistent with the suggestion that AIBP specifically targets lipid rafts modified by inflammatory or pathological agents and reduces raft abundance to normal levels.

### AIBP inhibits fusion between HIV-1 and target cells.

**FIG 3:**
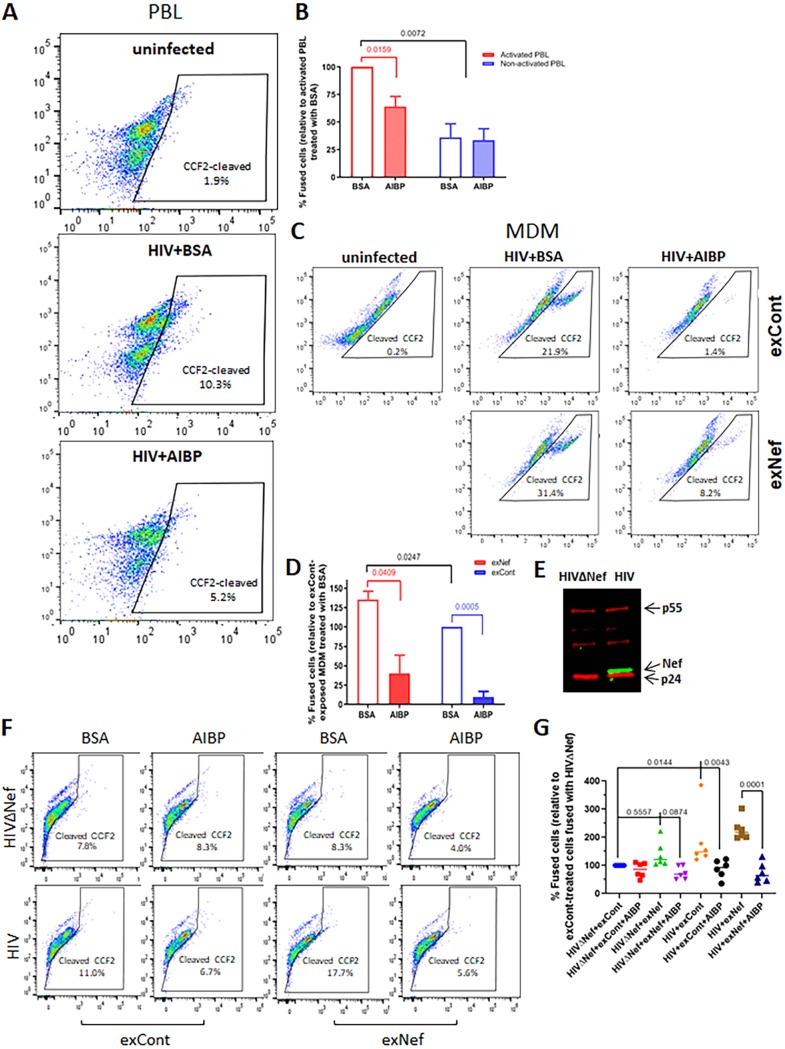
AIBP inhibits HIV fusion with target cells. (A) PBLs were activated with PHA for 48 h, treated with 0.2 μg/ml recombinant AIBP (or BSA as a control) for another 48 h, and exposed to BlaM-Vpr carrying HIV-1_NL4-3_ in the presence or absence of recombinant AIBP. Percentages of fused cells (cleaved CCF-2) were determined by flow cytometry. (B) Results of fusion analysis of PBLs from 4 donors (means ± SD) are presented relative to fusion of activated PBLs treated with BSA, taken as 100%. *P* values were calculated by multiple *t* test with Bonferroni-Dunn adjustment (BSA versus AIBP groups) or one-way ANOVA with Tukey’s adjustment (comparison of individual treatments); only significant values are shown. (C) MDMs were exposed to control exosomes (exCont) or Nef exosomes (exNef) for 48 h in the presence of 0.2 μg/ml recombinant AIBP (or BSA as a control) and were then infected with BlaM-Vpr carrying HIV-1_NL(AD8)_ in the presence or absence of recombinant AIBP. Percentages of fused cells were determined as described for panel A. (D) Results of fusion analysis of MDMs from 3 donors (means ± SD) are presented relative to fusion of exCont-exposed cells treated with BSA, taken as 100%. *P* values were calculated by multiple *t* test with Bonferroni-Dunn adjustment for multiple comparisons. (E) Western blot for Nef (green) and p55 (red) of HEK293T cells transfected with vectors expressing Nef-positive and Nef-negative HIV-1. (F) MDMs were exposed to exCont or exNef as described for panel C and infected with BlaM-Vpr carrying Nef-positive or Nef-deficient HIV-1. Fusion was analyzed as described for panel A. (G) An experiment was performed as described for panel F with MDMs from 6 donors. Results are presented for each donor relative to fusion of exCont-treated MDM with HIVΔNef. *P* values were calculated by repeated-measures ANOVA with Bonferroni correction for multiple comparisons.

Virus-cell fusion was analyzed by fluorescent HIV-1 virion-based assay (45). For the assay performed with PBLs, we used the CXCR4-tropic HIV-1 NL4.3 virus containing BlaM-Vpr. This analysis demonstrated that AIBP inhibits fusion between HIV-1 and PHA-activated PBLs (Fig. 3A; see also Fig. S4A). The inhibitory effects were consistent between cells from different donors, were statistically significant (*P* = 0.0159), and showed an average of 40% ± 10% inhibition (Fig. 3B). Fusion between HIV-1 and nonactivated PBLs was much less effective (approximately 70% less effective than that seen with PHA-activated cells) and was not inhibited by AIBP (Fig. 3B). We then analyzed the effect of AIBP on HIV-1 fusion with macrophages treated or not with exNef. For this assay, we used the CCR5-tropic HIV-1 pNL(AD8) (46). As expected, AIBP significantly (*P* = 0.0409) inhibited fusion with HIV-1 of MDMs treated with exNef (Fig. 3C and D). The level of fusion with HIV-1 of MDMs treated with exCont was significantly lower (*P* = 0.0247), but, surprisingly and in seeming contradiction to the effects on lipid rafts (Fig. 2D), was also significantly suppressed by AIBP (*P* = 0.0005) (Fig. 3C and D; see also Fig. S4B). The effects were consistent between the donors and averaged over 80% inhibition for cells treated with exCont and over 70% for cells treated with exNef (Fig. 3D). A possible explanation for this inconsistency is that Nef within HIV virions, or exNef contaminating virion preparations (47, 48), modified lipid rafts on target cells, making them susceptible to AIBP. Of note, fusion inhibitor T-20 (1 μg/ml) inhibited fusion by over 90% (Fig. S5).

10.1128/mBio.02956-19.4 FIG S4 Gating strategy for Fig. 3. (A) Gating for PBL (Fig. 3A). (B) Gating for MDM (Fig. 3C). (C) Gating for MDM (Fig. 3F). Download FIG S4, PDF file, 0.7 MB. Copyright © 2020 Dubrovsky et al. 2020 Dubrovsky et al. This content is distributed under the terms of the Creative Commons Attribution 4.0 International license.

10.1128/mBio.02956-19.5 FIG S5 Analysis of HIV fusion with MDM. (A) MDMs were treated with control exosomes for 48 h in the presence of 0.2 μg/ml recombinant AFP or AIBP (both proteins expressed from baculovirus vector) and then infected with BlaM-Vpr carrying HIV-1 NL(AD8) in the presence of AFP, AIBP, or 1 μg/ml T-20. Percentages of fused cells (cleaved CCF-2) were determined by flow cytometry. (B) Gating strategy. (C) Fusion analysis, performed as described for panel A, with MDMs from 3 donors. Results (mean ± SD) are presented relative to HIV fusion with cells treated with AFP, taken as 100%. Download FIG S5, PDF file, 0.7 MB. Copyright © 2020 Dubrovsky et al. 2020 Dubrovsky et al. This content is distributed under the terms of the Creative Commons Attribution 4.0 International license.

To test the possibility that Nef makes MDM-HIV fusion susceptible to AIBP-mediated inhibition, we compared the effects of AIBP on fusion between MDMs, exposed to exCont or exNef, and Nef-positive (HIV) or Nef-deficient (HIVΔNef) HIV-1 (Fig. 3E). The assay was performed with the CCR5-tropic NL4-3 constructs carrying recombinant gp120 with the CCR5-targeting V3 loop (49). Consistent with results shown in Fig. 3C and D, AIBP inhibited fusion of exCont-treated MDM with Nef-positive HIV-1 (Fig. 3F; see also Fig. S4C). It is important that fusion with this virus was less efficient than that seen with pNL(AD8), which carries a full envelope of the CCR5-tropic virus (compare results in Fig. 3F and C), and the observed differences were relatively small. We therefore confirmed these findings using MDMs from 5 more donors (total *n* = 6). Fusion of MDMs with HIVΔNef was consistently increased by treatment with exNef relative to exCont treatment (Fig. 3G), but the differences were not statistically significant (*P* = 0.5557). However, fusion of MDM with Nef-positive HIV was significantly more efficient than with HIVΔNef (*P* = 0.0144). This result appears to contradict two published reports that did not find any effect of Nef on fusion (50, 51). A likely explanation is that those studies were done with T cell lines or PHA-activated CD4^+^ T cells, which have high levels of lipid rafts (Fig. 2A) that are not influenced by Nef. AIBP did not affect fusion of exCont-treated MDMs with HIVΔNef. However, fusion with HIVΔNef of MDMs exposed to exNef showed a trend of being reduced by AIBP, although the difference did not reach significance (*P* = 0.0874). Fusion of MDMs with Nef-positive HIV-1 was inhibited by AIBP regardless of the presence or absence of exNef (Fig. 3G). Therefore, the effect of AIBP on HIV-1–macrophage fusion is dependent on the presence of Nef, either carried by the virus or delivered by exosomes.

Taken together, these results indicate that AIBP inhibits HIV infection by suppressing virus-cell fusion.

### Anti-HIV effect of AIBP *in vivo*.

**FIG 4:**
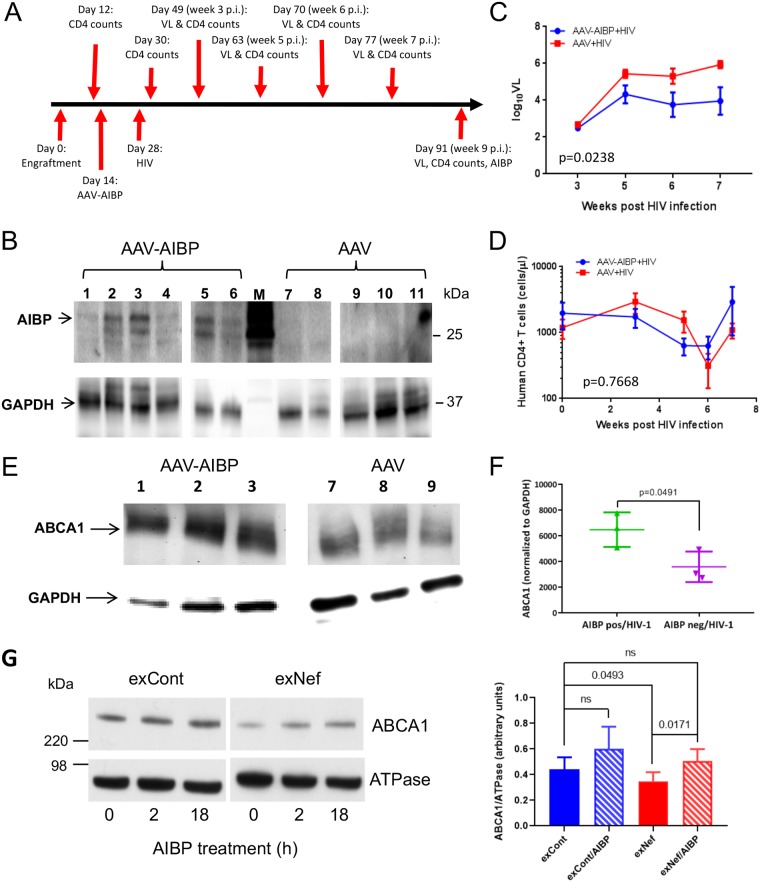
AIBP reduces HIV load and reverses ABCA1 downregulation in liver cells. (A) Timeline of the *in vivo* experiment. (B) Western blot analysis of livers from hu-mice infected with AAV and AAV-AIBP. (C) Viral load analysis of hu-mice. *, *P* = 0.0238 (by a 2-way ANOVA). (D) Analysis of human CD4^+^ cells in hu-mice. No significant differences were detected by 2-way ANOVA. (E) Western blot for AIBP and GAPDH (glyceraldehyde-3-phosphate dehydrogenase) in livers from hu-mice infected with HIV-1 and AAV-AIBP (mice 1, 2, and 3) and hu-mice infected with HIV-1 and empty AAV (mice 7, 8, and 9). (F) Quantitation of the blot in panel E. *P* values were calculated by unpaired *t* test. (G) HepG2 cells were treated for 48 h with exCont or exNef and were then incubated for 18 h in the presence or absence of AIBP (0.2 μg/ml). Total ABCA1 and K,Na ATPase (loading control) levels were assayed by Western blotting (left panel), and images from 6 independent experiments were quantified by ImageJ (right panel). Results present means ± SD. Significance was calculated by 1-way ANOVA with Tukey’s multiple-comparison adjustment. ns, not statistically significant.

To investigate the anti-HIV effects of AIBP in the *in vivo* setting, we used humanized mice (hu-mice). Immunodeficient mice were reconstituted with human CD4^+^ memory T lymphocytes (Table 1). One group of hu-mice (animals 1 to 6) was injected intravenously with nonreplicating adeno-associated virus (AAV) vector expressing His-tagged AIBP. As a control, another group (animals 7 to 12) was injected with empty AAV. Two weeks after AAV injection, all hu-mice were infected with HIV-1 ADA using intraperitoneal injection. We used the R5 HIV-1 strain here to mimic human infections, which are transmitted almost exclusively with CCR5-tropic viruses (52). Mice were maintained for 9 weeks after HIV infection; after that period, mice were sacrificed and AIBP expression was measured by Western blotting of the liver tissue (direct detection of AIBP in blood is challenging, likely due to rapid binding of AIBP to cells [5, 7, 8]). The timeline of the experiment is presented in Fig. 4A.

**TABLE 1:** Reconstitution of mice with human CD4^+^ memory T cells^*a*^

Mouse ID	No. of human CD4^+^ memory T cells/μl
1	1,117
2	990
3	1,086
4	1,024
5	6,265
6	1,342
7	790
8	611
9	1,122
10	1,458
11	235
12	2,923

a Cells were analyzed by flow 2 weeks after reconstitution. ID, identifier.

All hu-mice injected with AIBP-AAV expressed His-tagged AIBP in the liver (Fig. 4B). The AAV-DJ/8 vector used in this study has a mutation in the heparin binding domain, which lifts the liver restriction of AAV-DJ and expands its transduction to nonhepatic tissues (53). Detection of the His-tagged AIBP indicates that AAV-mediated expression was stable and continued throughout the duration of the experiment. Analysis of HIV-1 load revealed lower HIV levels of replication in AIBP-expressing mice (Fig. 4C), and two-way analysis of variance (ANOVA) demonstrated that the differences between the groups were statistically significant. One mouse (animal 5) in the AAV-AIBP group did not get infected by HIV-1 at all, despite the fact that it was effectively reconstituted with human T cells (Table 1). No statistically significant differences between the groups were found in the numbers of human CD4^+^ T cells (Fig. 4D).

Our previous study demonstrated a reduction of ABCA1 abundance in livers of simian immunodeficiency virus (SIV)-infected monkeys (18). The abundance of ABCA1 was significantly higher (*P* = 0.0491) in HIV-infected animals expressing AIBP than in HIV-infected mice exposed to empty AAV (Fig. 4E and F). Of note, similarly to previous observations in SIV-infected macaques (18), suppression of ABCA1 in HIV-infected untreated mice was not complete, likely due to compensatory upregulation of ABCA1 mRNA expression (13). To further evaluate this activity of AIBP, we tested whether it can protect human hepatocytes from ABCA1 downregulation induced by exNef. Human HepG2 cells were treated with exCont or exNef (equalized by protein content) for 18 h in the presence or absence of recombinant AIBP (0.2 μg/ml) and ApoA-I (50 μg/ml), and levels of ABCA1 and K,Na ATPase (loading control) were assessed by Western blotting (Fig. 4G, left panel). Bands were quantified by the use of ImageJ, and the ABCA1/ATPase ratio was calculated for 5 independent experiments (Fig. 4G, right panel). This analysis confirmed that exNef significantly downregulated ABCA1 (*P* = 0.0493) and that AIBP reversed this effect (*P* = 0.0171). Taken together, these results indicate that AIBP not only reduces HIV replication but also protects host cells from indirect effects of HIV infection, which are likely mediated by the factors, including exNef, released from HIV-infected cells (18).

### HLA-B genotype influences the anti-HIV activity of AIBP.

**FIG 5:**
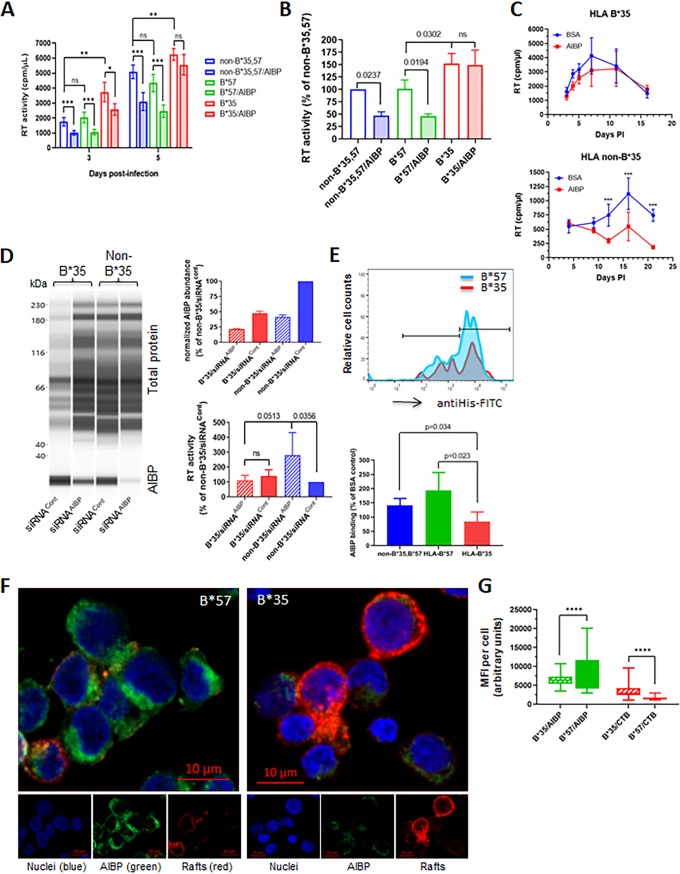
Anti-HIV effect of AIBP is reduced in cells from HLA-B*35 donors. (A) PHA-activated PBMCs from donors with HLA-B*35, HLA-B*57, and non-B*35,B*57 genotypes were infected with HIV-1_LAI_ and incubated in the presence or absence of recombinant AIBP. Virus replication was followed by analysis of RT activity. Results are presented for donors B*35/55 (B*35), B*51/57 (B*57), and B*27/38 (non-B*35,B*57). Results are presented as means ± SD of results from 5 replicates. *, *P* = 0.01; **, *P* < 0.01; ***, *P* < 0.001 (by multiple-comparison tests with Holm adjustment). (B) The experiment was performed as described for panel A with cells from 3 donors of each genotype. Results (means ± SD) are presented relative to cells from non-B*35,57 donor (taken as 100%) at the time point corresponding to the peak of infection (day 5 p.i.). *P* values were calculated by ordinary one-way ANOVA with Tukey’s multiple-comparison adjustment. (C) PHA-activated PBMCs from HLA-B*35 or non-B*35 donors were infected with T/F strain pCH185.c/K3016 and cultured in the presence or absence of 0.2 μg/ml recombinant AIBP or BSA. Virus replication was followed by analysis of RT activity. Results show means ± SD (*n* = 4). Significance was calculated by multiple *t* tests with Holm-Sidak correction for multiple comparisons. ***, *P* < 0.001. (D) PHA-activated PBMCs from HLA-B*35 and non-B*35 donors were treated with AIBP-targeting (siRNA^AIBP^) or control (siRNA^Cont^) Accell siRNA. AIBP abundance was measured by ProteinSimple Western blotting (left panel) and normalized against total protein, and results are presented relative to non-B*35 cells treated with siRNA^Cont^ (right top panel). Cells were infected with HIV-1 LAI, and RT activity in culture supernatant was measured on day 4 postinfection (right bottom panel). Results show means ± SD of 4 replicates. Ordinary one-way ANOVA with Tukey’s adjustment for multiple comparisons was used to calculate *P* values. (E) (Top panel) Binding of recombinant AIBP to cells with different HLA-B genotypes was analyzed by flow cytometry using anti-His antibody. (Bottom panel) Quantitation of AIBP binding to cells from 4 different donors each of genotypes B*35 and non-B*35,B*57 and 3 donors of genotype B*57. *P* values were calculated by multiple *t* tests, with *post hoc* Holm adjustment for multiple comparisons. (F) Binding of recombinant AIBP to cells with HLA-B*35 and HLA-B*57 genotype was analyzed by fluorescence microscopy using Alexa Fluor 555-conjugated CTB for lipid rafts (red), FITC-conjugated anti-His antibody for AIBP (green), and DAPI for nuclei (blue). (G) Quantification of MFI on 108 cells with HLA-B*35 and 159 cells with HLA-B*57 genotype using Volocity software. ****, *P* < 0.0001 (calculated by multiple *t* tests with Holm-Sidak correction for multiple comparisons).

The impact of host genetic variation on susceptibility to HIV infection and progression of the disease has been well established (54). In particular, specific *HLA* alleles have been found to be the primary determinants of the rate of progression to AIDS (55–58). For example, the HLA-B*35 genotype has been shown previously to be associated with rapid progression of the disease (55), whereas the HLA-B*57 allele has been consistently associated with slower disease progression (59). This has been explained by differences between the alleles in antigenic peptide presentation (60). However, given the association of major histocompatibility complex class I (MHC-I) molecules with lipid rafts (61), which are regulated by AIBP, we hypothesized that HLA-B genotype may also influence HIV replication by altering the effect of AIBP. We infected peripheral blood mononuclear cells (PBMCs) isolated from 3 donors with HLA-B*35, HLA-B*57, and non-B*35,57 genotypes with HIV-1 LAI and followed virus replication in the presence or absence of recombinant AIBP. As shown in Fig. 5A, the suppressive effect of AIBP on HIV replication was significantly lower in cells from HLA-B*35 donors on day 3 postinfection and had completely disappeared by day 5 p.i., whereas AIBP-mediated suppression was still highly significant (*P* < 0.001) in two other donors with non-B*35 genotype. No significant differences in anti-HIV activity of AIBP were found between B*57 and non-B*35,57 donors, suggesting that the relative resistance of HLA-B*57-positive people to HIV disease progression is not due to AIBP sensitivity. Importantly, HIV-1 replication in cells from HLA-B*35 donor was significantly higher than in cells from B*57 and non-B*35,57 donors (Fig. 5A). These results were confirmed using cells from 3 donors with each of B*35, B*57, and non-B*35,57 genotypes (Fig. 5B). On day 5 postinfection, AIBP was seen to have significantly downregulated HIV-1 replication in donors with non-B*35 genotype but not in HLA-B*35 donors. HIV-1 replication levels in cells from B*35 donors were higher than in cells with non-B*35 genotype, although the differences were relatively small (*P* = 0.0302). No significant differences were observed between cells with HLA-B*57 and non-B*35,57 genotypes in HIV-1 replication or susceptibility to AIBP suppression (Fig. 5B). We further extended this observation to the T/F HIV-1 strain pCH185.c/K3016 (31). Again, recombinant AIBP reduced replication of the T/F virus in cells from non-B*35 donor but not in cells from HLA-B*35 donor (Fig. 5C). Strikingly, knockdown of endogenous AIBP significantly increased HIV-1 LAI replication in cells from non-B*35 donor but did not affect replication in HLA-B*35 cells (Fig. 5D, bottom right panel). We next measured the binding of recombinant AIBP to cells with different HLA-B genotypes. AIBP binding to cells from HLA-B*35 donors was significantly lower than binding to cells from HLA-B*57 donors or from donors with non-B*35,57 genotype (Fig. 5E; see also Fig. S6). Again, the differences between B*57 and non-B*35,57 donors were not significant (Fig. 5E, bottom panel). The differences in AIBP binding are illustrated in Fig. 5F, where cells from HLA-B*35 and HLA-B*57 donors are shown, and are quantified in Fig. 5G. Cell-bound His-tagged AIBP was brightly stained on cells from HLA-B*57 donor and was much less brightly stained on cells from HLA-B*35 donor (Fig. 5F). The opposite was observed with CTB staining, which reflects abundance of lipid rafts; rafts were much more brightly stained on HLA-B*35 cells than on B*57 cells, consistent with the proposed raft-reducing activity of AIBP. Quantitation of AIBP binding demonstrated significantly reduced binding to HLA-B*35 cells, whereas the levels of lipid raft staining were significantly higher on HLA-B*35 cells than on HLA-B*57 cells (Fig. 5G). These results indicate that HLA-B*35 genotype impairs AIBP binding to target cells and makes cells insensitive to the anti-HIV activity of AIBP, both endogenously produced and added exogenously.

10.1128/mBio.02956-19.6 FIG S6 Gating strategy for Fig. 5E. Download FIG S6, PDF file, 0.6 MB. Copyright © 2020 Dubrovsky et al. 2020 Dubrovsky et al. This content is distributed under the terms of the Creative Commons Attribution 4.0 International license.

## DISCUSSION

Accumulating evidence suggests a protective and, possibly, a therapeutic role of AIBP in human diseases associated with inflammation and impairment of cholesterol metabolism, in particular, in atherosclerosis (6, 62). The main finding of this study is that AIBP also exerts anti-HIV activity. Mechanistically, AIBP decreased the abundance of lipid rafts on activated cells, reducing virus-cell fusion. Importantly, no change in the abundance of lipid rafts or fusion was observed after incubation of nonactivated cells with AIBP. This result is consistent with previously reported findings and suggests that AIBP specifically targets lipid rafts on cells subjected to an inflammatory or infectious agent or factors produced by infected cells (6, 7). One such “activating” factor could be Nef-containing exosomes (exNef). Indeed, AIBP reversed the effect of exNef on the abundance of lipid rafts on MDMs.

Our results suggest that exNef, which are produced by HIV-infected cells even in the presence of suppressive antiretroviral therapy (ART) (63, 64), may enhance HIV-associated pathology by manipulating lipid rafts on uninfected cells. Nef is considered the key pathogenic factor of HIV due to its profound effects on viral replication, immune systems, and multiple tissues (65–67). Previous studies attributed the pathogenic activity of Nef to its ability to suppress cytotoxic T lymphocyte (CTL) responses by downregulating MHC-I (68) and to stimulate viral spread by downregulating CD4 (69) and SERINC3 and SERINC5 in infected cells (70, 71). Our findings suggest another previously unappreciated activity of Nef: Nef exosomes increase the abundance of lipid rafts on macrophages, stimulating HIV infection (this report) and potentiating lipid raft-dependent inflammatory responses (42).

Our finding that AIBP inhibits fusion of the Nef-positive virus, which does not incorporate SERINC (70, 71), but does not inhibit fusion of Nef-deficient virus, which incorporates SERINC, suggests that fusion of SERINC-positive HIV occurs via lipid rafts that are insensitive to AIBP. One suggested mechanism of SERINC5 anti-HIV activity is that it forms large oligomers, which harden the viral membrane, restrict lipid diffusion, slow the folding of the envelope for fusion, and decrease virus-cell fusion (72, 73). The slowed fusion of such virus may proceed via “normal” lipid rafts, which are not affected by AIBP. However, treatment of MDMs with exNef induces formation of “pathological” rafts, increases fusion with Nef-deficient HIV, and makes fusion susceptible to AIBP inhibition. Interestingly, pretreatment of macrophages with exNef, while increasing abundance of lipid rafts, did not increase MDM fusion with wild-type HIV, suggesting that Nef delivered either by virions or by virion-contaminating exosomes is sufficient to ensure maximal fusion. This effect of Nef on fusion contradicts previous reports that concluded that Nef does not alter fusion (50, 51). The difference with those studies is that the effects reported here were observed with MDMs, whereas the reports cited above used T cell lines or PHA-activated CD4^+^ T cells. Our analysis demonstrated that activated T cells had high levels of lipid rafts (Fig. 2A) that were not much changed by adding exNef and, relative to MDMs, had very high levels of endogenous AIBP expression (Fig. 1H) that may have masked the effect of Nef. It remains to be tested whether exNef increases the abundance of lipid rafts on nonactivated T cells and whether this increases levels of fusion with HIV.

In this study, we used a simplified model of humanized mice, consisting of immunodeficient mice reconstituted with human memory CD4^+^ T lymphocytes. This model allows avoidance of graft versus host reaction (74) and enables maintenance of HIV replication for several months. The lack of human myeloid cells in this model is a limitation, especially in view of the role of these cells in HIV infection (75) and of their sensitivity, relative to CD4^+^ T lymphocytes, to agents targeting cholesterol efflux (17, 27, 76). *In vitro* experiments with MDMs performed in this study demonstrated that AIBP potently inhibits HIV replication in these cells, so we expect that AIBP would exert even more pronounced anti-HIV activity in a model containing a full range of HIV-susceptible cells.

AAV-delivered AIBP was detected in liver lysates (Fig. 4B), but the levels in plasma were undetectable despite the fact that the AIBP construct was designed to produce a secreted protein. This agrees with a previous report that AIBP was undetectable in normal human plasma (1). In addition, AIBP was not detected in any HDL proteomic studies despite its documented binding to ApoA-I and HDL (1, 5). This can be explained by the fact that secreted AIBP binds to inflammatory cells (7, 8) and to activated PBLs (Fig. 5), leading to rapid clearance from plasma of the AIBP secreted from liver and other tissues in AAV-AIBP-infected mice.

The finding that HLA-B*35 genotype was associated with reduced AIBP binding and with decreased anti-HIV activity was serendipitous. Given that HLA association with lipid rafts has been previously suggested (61), it is likely that HLA-B*35-dependent modifications of lipid raft structure/composition influence AIBP binding to cells, modulating its ability to reduce lipid rafts and inhibit virus-cell fusion. This may be a factor contributing to the known association of HLA-B*35 genotype with fast disease progression. This conclusion is supported by our finding that AIBP silencing did not affect HIV-1 replication in the cells with HLA-B*35 genotype whereas it did significantly increase virus replication in cells with other HLA genotypes. The HLA-B*35 genotype appears to stand out, as we did not find significant differences in AIBP binding or anti-HIV activity between cells of other genotypes tested in this study. It remains to be established whether other HLA genotypes associated with HIV susceptibility or control influence AIBP binding before more-elaborate mechanistic studies of HLA-mediated changes in lipid rafts are initiated.

The mechanism of the anti-HIV activity of AIBP is likely to involve its ability to reduce the abundance of lipid rafts. This conclusion is based on the well-established capacity of AIBP to disrupt lipid rafts (1, 5, 7, 8) and on the role of rafts in HIV fusion (33); concurrent effects of AIBP on rafts and HIV fusion were demonstrated in several independent experimental systems throughout this study. The mechanism behind AIBP-mediated disruption of lipid rats was not investigated in this study, but previous reports suggested that AIBP stimulates cholesterol efflux, depleting rafts of cholesterol (1, 5). It may also stabilize ABCA1 (9), providing additional capacity for cholesterol efflux.

Results of this study demonstrate that AIBP is an innate anti-HIV restriction factor. Although the pathways regulating endogenous AIBP expression and secretion are not well understood, existing evidence points to spatiotemporal and/or regulated patterns of AIBP secretion (1, 5, 8). As described in this study, low sensitivity to AIBP-mediated anti-HIV activity of cells from HLA-B*35 donors, which are susceptible to fast progression of HIV disease, suggests the role of AIBP in controlling natural HIV infection. In this work, we sought to augment the benefit of an innate AIBP protective mechanism by delivering recombinant protein or AAV-expressed AIBP to target activated host cells and make them less susceptible to HIV infection. The anti-HIV effect of such treatment, while statistically significant, was relatively small compared to the effects of antiretroviral drugs (e.g., T-20). One potential reason for the low activity of exogenously added AIBP is that its effect was measured on the background of endogenous AIBP. PBLs are hard to transfect, and the silencing approach that we used produced only a 30% to 50% downregulation of endogenous AIBP levels. Future studies with engineered AIBP-deficient cells are likely to produce more impressive results. Given that lipid rafts are used by many pathogens as an entry platform (77), AIBP may also protect against infection by other viruses and microbes. Overall, this study revealed a novel innate factor that inhibits HIV infection by targeting lipid rafts.

## MATERIALS AND METHODS

### Cells and HIV infection.

Peripheral blood mononuclear cells (PBMCs) were isolated from whole blood (purchased from NY Blood Bank) by Ficoll gradient centrifugation. Monocyte-derived macrophages (MDMs) were prepared from PBMCs by plastic adherence and differentiation for 7 days in the presence of 50 ng/ml macrophage colony-stimulating factor (M-CSF; Sigma) as previously described (78). HIV-1 ADA was used for infection of MDMs. Nonadherent cells (peripheral blood lymphocytes [PBLs]) were activated or not with PHA (5 μg/ml) and interleukin-2 (IL-2) (20 U/ml) for 2 days prior to infection with HIV-1 LAI or primary transmitted/founder (T/F) virus, the CCR5-tropic strain pCH185.c/K3016 (31).

### Recombinant AIBP.

AIBP was produced in a baculovirus/insect cell system to allow posttranslational modifications and to ensure endotoxin-free preparations. Human AIBP was cloned into a pAcHLT-C vector behind the polyhedrin promoter. The vector contains an N-terminal His tag to enable purification and detection. Insect Sf9 cells were transfected with BD BaculoGold baculovirus DNA and the AIBP vector to produce a baculovirus stock. Fresh Sf9 cells were infected with the AIBP-producing baculovirus, cell pellets were collected after 3 days, and His-AIBP was purified on a nickel-nitrilotriacetic acid agarose column. Protein was dialyzed against saline solution, and aliquots were stored at –80°C. AIBP was used at a concentration of 0.2 μg/ml unless otherwise indicated.

### Analysis of AIBP binding to cells by flow cytometry.

PBMCs from donors with the following HLA-B genotypes were purchased from AllCells Inc.: B*35 positive (B*15:17:01/B*35:01:01, B*35:01:01/B*55:01:01, B*35:01:01/B*35:01:01, B*35:08:01/B*51:01:01); B*57 positive (B*07:02:01/B*57:01:01, B*51:01:01/B*57:01:01, B*40:01:02/B*57:01:01); non-B*35,B*57 (B*08:01:01/B*38:01:01, B*27:05:02/B*38:01:01, B*07:02:01/B*27:05:02, B*15:11:01/B*51:01:02). To analyze AIBP binding, PBMCs were blocked with Tris-buffered saline (TBS) containing 1% BSA for 30 min on ice and incubated with either 2 μg/ml BSA or recombinant His-tagged AIBP for 2 h on ice. Cells were washed with phosphate-buffered saline (PBS) and incubated with 1 μg/ml fluorescein isothiocyanate (FITC)-conjugated anti-His polyclonal antibody (Abcam) and LIVE/DEAD Fixable Aqua dead cell stain (Invitrogen) for 1 h at 4°C. After washes with PBS, cells were analyzed by flow cytometry gating on live cells.

### Analysis of AIBP binding to cells by fluorescence microscopy.

PBMCs from HLA-B*35 and HLA-B*57 donors were incubated with His-AIBP as described above and stained for bound AIBP with FITC-conjugated anti-His polyclonal antibody (Abcam) and for lipid rafts with Alexa Fluor 555-conjugated cholera toxin subunit B (CTB). Cells were then mounted on microscopic glass slides, fixed, and permeabilized with Triton X-100, and nuclei were stained with DAPI (4′,6-diamidino-2-phenylindole). Imaging and analysis were performed on a Cell Observer spinning-disk fluorescence microscope (Carl Zeiss) equipped with a Yokogawa CSU X1 spinning disk and Evolve Delta electron microscopy (EM) charge-coupled-device (CCD) cameras (Photometrics) (512 by 512 pixels). A Plan Apochromat 63×/1.46 oil lens objective was used to visualize the optical section close to the center of the majority of the cells. The camera exposure time for each channel and the emission and excitation parameters were kept constant across the experiments. DAPI was excited with a 405 diode laser, and the emission was recorded with a 450/50 bandpass filter. FITC was excited with a 488 diode laser, and emission was recorded with a 535/30 bandpass filter. A 561 diode laser was used for excitation to record the CTB immunolabeling, and the emission was recorded with a 629/62 emission filter. Images were further enhanced using ZEN Microscope Software (Carl Zeiss). Again, identical settings were used for all images. The final images were saved as TIFF files.

Mean fluorescence intensity (MFI) quantitation was performed on 108 individual cells with HLA-B*35 genotype and 159 cells with HLA-B*57 genotype. To compute the cellular intensities for AIBP and CTB (lipid rafts), the original data stored as czi files (the Carl Zeiss Image Data file type) were used. In each image, taken with a 63× objective, every cell was evaluated using Volocity software. Individual cells were outlined using the freehand region of interest (ROI) tool, and each cell’s overall cellular fluorescent intensity was recorded for each channel. Outliers were removed using statistical software in the GraphPad Prism 8 program.

### Isolation and purification of exosomes.

At 48 h posttransfection of HEK293T cells (purchased from ATCC) with pcDNA3.1 vector expressing Nef of HIV-1 NL4-3 (to make exNef) or with empty vector (to make exCont), medium was collected from cell cultures. Exosomes were isolated by differential centrifugation, as described previously (79). Briefly, culture supernatants were preclarified by centrifugation at 500 × *g* for 10 min at 4°C to remove cells and cellular debris and were then clarified by spinning at 2,000 × *g* for 30 min at 4°C to remove the remaining debris and large apoptotic bodies, and exosomes were pelleted by centrifugation at 100,000 × *g* for 75 min at 4°C. The pellet was resuspended in exosome-free medium and frozen at **–**70°C. Of note, the pellet contained a mixture of extracellular vesicles that were 25 to over 150 nm in size (see Fig. S1 in the supplemental material), corresponding to exosomes, microvesicles, and other vesicles (80). However, since the majority of vesicles had the size of 150 nm that is characteristic of exosomes, we use the term “exosomes” in this report. Total protein content in exosome samples was estimated by Bradford assay after dilution in radioimmunoprecipitation assay (RIPA) buffer and boiling for 3 min; A 1-μg volume of exosomes was used to treat 1 × 10^6^ cells.

10.1128/mBio.02956-19.1 FIG S1 Visualization of extracellular vesicles (EVs) with flow cytometry. (A) Defining sizing gates with Megamix beads. Fluorescent Megamix-plus SSC beads were used according to the instructions of the manufacturer (Cosmo Bio, CA). (B) EV visualization with flow cytometry. exCont and exNef EVs were labeled with the lipophilic tracer BODIPY (Invitrogen, Life Technologies, CA) and visualized with a LSR II flow cytometer (Becton Dickinson) as BODIPY-positive events thresholding on BODIPY fluorescence. (Left column) Gating strategy for flow analysis of BODIPY-labeled EVs isolated from mock-transfected (upper panel) or Nef-transfected (lower panel) HEK293T cells. A singlet gate was defined by plotting fluorescence height versus fluorescence width. The gate excludes events with a high width and high height that represented aggregates. (Right column) EV sizing as defined by Megamix-plus SSC bead gates (A). Results represent one of two similar experiments. In each plot, the fractions of total events in their respective gates are shown. Download FIG S1, PDF file, 0.7 MB. Copyright © 2020 Dubrovsky et al. 2020 Dubrovsky et al. This content is distributed under the terms of the Creative Commons Attribution 4.0 International license.

### Nanoparticle tracking analysis of exosomes.

The size and the concentration of exosomes were determined using a NanoSight NS300 instrument (Malvern Instruments Ltd., Malvern, United Kingdom) based on nanoparticle tracking analysis (NTA). NTA utilizes the properties of both light scattering and Brownian motion to obtain the particle size distribution of samples in liquid suspension. Briefly, exosome samples from Nef-transfected and mock (empty vector)-transfected HEK293T cells were diluted 1:100 in PBS, and exosomes were tracked on the NanoSight NS300 instrument. The samples were loaded by means of the use of a constant pressure syringe pump controller. Videos were recorded for 60 s two times, at camera setting 13, and were analyzed with NTA software 3.0 (Malvern instruments Ltd., Malvern, United Kingdom).

### Fusion assay.

The fluorescence HIV-1 virion-based assay was used as previously described (45). The CCF2 substrate was purchased from Life Technology and added to cells at a final concentration of 1 μM. The CCR5-tropic viruses used for MDMs were as follows: (i) pNL(AD8), which carries Env of strain AD8 (46), used at 1 × 10^6^ cpm of reverse transcriptase (RT) activity per 10^6^ cells; (ii) pBRNL4.3_92BR020.4(R5)nef−_IRES_GFP and pBRNL4.3_92BR020.4(R5)nef+_IRES_GFP (49), the Nef-negative and Nef-positive recombinant constructs, respectively, both used at 2 × 10^6^ cpm of RT activity per 10^6^ cells. The CXCR4-tropic virus for PBLs was pNL4-3 used at 1 × 10^6^ cpm of RT activity per 10^6^ cells. Cells were analyzed by flow cytometry; the proportion of cells showing fluorescence at 450 nm characteristic of the cleaved CCF2 reflected the percentage of cells fused with HIV.

### Lipid raft analysis.

The abundance of lipid rafts was evaluated by binding of cholera toxin subunit B (CTB), as previously described (17). Briefly, cells were incubated for 1 h at 4°C in serum-containing medium with FITC-CTB conjugate (Invitrogen) (final concentration, 0.5 μg/ml), fixed with 5% formaldehyde, and analyzed by flow cytometry gating on live cells as revealed by a LIVE/DEAD Fixable Aqua dead cell stain kit (Invitrogen).

### AAV-AIBP.

Murine AIBP was fused with fibronectin secretion sequence (FIB) at the N terminus and 6×His at the C terminus (FIB-AIBP-His). FIB-AIBP-His was cloned into pAAV-MCS vector (Agilent Technologies). AAV-293 cells (Agilent Technologies) were transfected with 20 μg each of pAAV-FIB-mAIBP-His, pAAV-DJ/8 (Cell Biolabs), and pHelper DNA (Cell Biolabs). Subsequent steps of virus harvest, purification, and storage were performed according to published protocols (81). Viral DNA was extracted from purified virus, and the number of gene copies (gc) was determined using quantitative PCR (qPCR) with primers for the inverted terminal repeats (TaKaRa Bio Inc.).

### AIBP silencing in PBLs.

Silencing was performed using Accell SMARTpool siRNA (4 siRNAs) and delivery mix (Dharmacon) following the manufacturer’s protocol. The AIBP target sequences were CGAGUGUGUCUAUCGUCUG, UGACGAUUGAUGAACUGUA, CUACUGUCCUGGUCAUCUG, and UCAGCGUGGACCAACUUAU.

### Western blotting.

Western blotting was performed on ProteinSimple Jess microfluidic capillary equipment, using the manufacturer’s software for band quantification. Unless indicated otherwise, the loading control for quantification was total protein measured in the same capillary as the protein of interest, using ProteinSimple proprietary technology. Nef was detected using anti-Nef rabbit polyclonal antibody from the NIH AIDS Reagent Program (82) followed by secondary goat anti-rabbit-Green antibody (ProteinSimple). For p55, we used anti-p24 mouse monoclonal antibody (AG3.0) from the NIH AIDS Reagent Program (83), followed by secondary goat anti-mouse-Red antibody (ProteinSimple). For AIBP detection, rabbit polyclonal anti-AIBP antibody from Novus Biologics was used, followed by anti-rabbit horseradish peroxidase (HRP) antibody from ProteinSimple.

### MTT assay.

Cell metabolic activity was measured by the use of an MTT assay kit (Abcam) following the manufacturer’s instructions.

### Humanized mice.

Memory CD4^+^ T-cells were isolated from PBMCs (Peripheral Blood Leuko Pak; AllCells) by negative selection (EasySep human memory CD4^+^ T cell enrichment kit; Stemcell Technologies) following the manufacturer’s instructions. Isolated memory CD4^+^ T-cells were then engrafted into 7-to-9-week-old NOD.Cg-*Prkdc^scid^ Il2rg^tm1wjl^*/SzJ (NSG) mice (The Jackson Laboratory) at 10^7^ cells per animal via tail vein injection. Peripheral blood was collected weekly postengraftment by tail nick to assess human cell reconstitution by flow cytometry and to measure HIV load (see below). At 2 weeks after CD4^+^ T-cell engraftment, mice were intravenously injected with empty virus or AAV-AIBP at 1 × 10^12^ gc/mouse and after 2 more weeks were infected with 70,000 50% tissue culture infective doses (TCID_50_)/animal of HIV-1 ADA virus via intraperitoneal injection. Animals were sacrificed at the study conclusion, and liver samples and peripheral blood were collected.

### Flow cytometry of cells from hu-mice.

Peripheral blood cells from hu-mice were resuspended in a staining cocktail of anti-human CD27 (clone O323; BioLegend), CD197 (clone G043H7; BioLegend), CD45RA (clone HI100; BD Biosciences), CD8a (clone RPA-T8; BioLegend), CD4 (clone RPA-T4; BD Biosciences), CD3 (clone SK7; BD Biosciences), and CountBright Absolute Counting Beads (for cell quantification; Thermo Fisher). Red blood cells (RBCs) were lysed in RBC lysis/fixation solution (BioLegend), and the remaining cells were fixed with 4% paraformaldehyde. Fixed cells were analyzed by flow cytometry on an LSRFortessa X-20 cell analyzer (BD).

### HIV load.

Viral RNA was extracted from cell-free plasma using a QIAamp viral RNA minikit (Qiagen) following the manufacturer’s instructions. HIV RNA was quantified by reverse transcriptase quantitative PCR (qRT-PCR) using the integrase single-copy assay (iSCA) (84). Reactions were performed with an AgPath-ID one-step RT-PCR kit (Applied Biosystems), using 400 nM primers (forward primer, 5′-TTTGGAAAGGACCAGCAAA-3′; reverse primer, 5′-CCTGCCATCTGTTTTCCA-3′) and 250 nM dually labeled probe (probe, 5′-FAM [6-carboxyfluorescein]-AAAGGTGAAGGGGCAGTAGTAATACA-TAMRA [6-carboxytetramethylrhodamine]-3′) targeting a highly conserved 127-bp region of the HIV integrase gene. Absolute quantifications were established by comparison to a standard curve of *in vitro* transcribed HIV-1 RNA standards generated by cloning the p31 region of *pol* from plasmid pNL4-3 containing an infectious clone of HIV-1 (GenBank accession number K02013). PCR was first used to generate a 418-bp amplicon from pNL4-3 by the use of 600 nM primers (forward primer, 5′-CCCTACAATCCCCAAAGTCA-3′; reverse primer, 5′-CACAATCATCACCTGCCATC-3′). The resulting amplicon was cloned into pGEM T-Easy vector (Promega) downstream of the T7 promoter. Plasmid containing the correct insertion was linearized with SacI, and *in vitro* RNA synthesis was performed using a 4-h incubation at 37°C and a MEGAscript T7 transcription kit (Thermo Fisher). Template DNA was degraded by treatment with RQ1 RNase-free DNase (Promega), and RNA was purified using an RNeasy minikit (Qiagen) followed by deoxynucleoside triphosphate (dNTP) removal (Qiagen). Purified RNA was quantified using spectrophotometry at 260 nm, diluted in a mixture containing 5 mM Tris, 1 μM dithiothreitol (DTT), and 1,000 units/ml of recombinant RNasin RNase inhibitor (Promega), and stored at –80°C until use.

### Statistical analysis.

The experiments were conducted in triplicate and repeated 2 to 5 times. The statistical significance of the differences was assessed (unless indicated otherwise) by ordinary one-way ANOVA with Tukey’s adjustment for multiple comparisons (for comparisons of 3 or more samples) or by *t* test with Bonferroni-Dunn adjustment for multiple comparisons (for repeated-measures comparisons of 2 samples) in GraphPad software package Prism 8. *P* values of <0.05 were considered significant.

### Ethics statement.

All animal procedures in this study were conducted under IACUC protocol A333 approved by The George Washington University in compliance with the Animal Welfare Act and in accordance with the principles set forth in the Guide for the Care and Use of Laboratory Animals.
