# Erratum for Dubrovsky et al., “Inhibition of HIV Replication by Apolipoprotein A-I Binding Protein Targeting the Lipid Rafts”

**DOI:** 10.1128/mBio.00234-20

**Published:** 2020-03-17

**Authors:** Larisa Dubrovsky, Adam Ward, Soo-Ho Choi, Tatiana Pushkarsky, Beda Brichacek, Christophe Vanpouille, Alexei A. Adzhubei, Nigora Mukhamedova, Dmitri Sviridov, Leonid Margolis, Richard B. Jones, Yury I. Miller, Michael Bukrinsky

**Affiliations:** a The George Washington University School of Medicine and Health Sciences, Washington, DC, USA; b University of California San Diego, La Jolla, California, USA; c Eunice Shriver National Institute of Child Health and Human Development, National Institutes of Health, Bethesda, Maryland, USA; d Engelhardt Institute of Molecular Biology, Russian Academy of Sciences, Moscow, Russia; e Baker Heart and Diabetes Research Institute, Melbourne, Victoria, Australia

## ERRATUM

Volume 11, no. 1, e02956-19, 2020, https://doi.org/10.1128/mBio.02956-19. Dr. Sviridov’s first name was spelled incorrectly in the original article. The byline should appear as shown above.

