# Supplementary material for: Inhibition of HIV Replication by Apolipoprotein A-I Binding Protein Targeting the Lipid Rafts
Source: mBio. 2020 Jan 21;11(1):e02956-19. doi: 10.1128/mBio.02956-19 (PMC6974568; doi:10.1128/mBio.02956-19)
Supplement: FIG S2 [file mBio.02956-19-sf002.pdf]

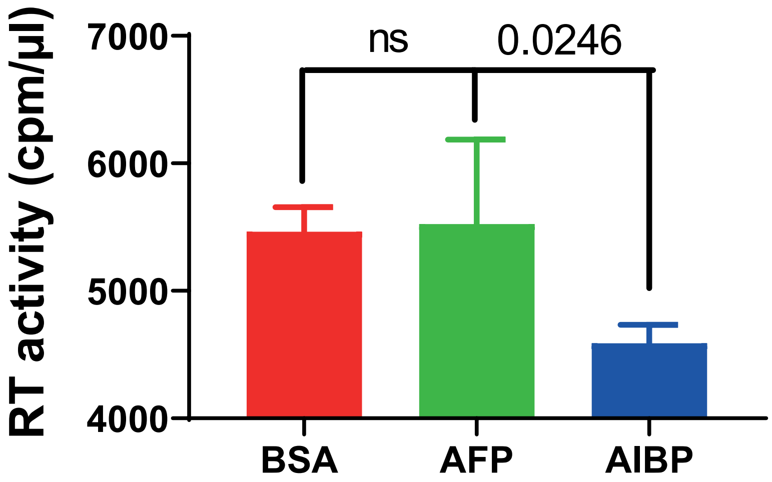

Figure S2. AIBP inhibits HIV-1 replication. PHA-activated PBMCs were infected in quadruplicate wells with HIV-1 LAI and cultured for 5 days in the presence of 0.2 µg/ml of BSA, baculovirus-expressed alpha-fetoprotein (AFP), or baculovirus-expressed AIBP. Virus production was measured by RT activity in culture supernatant. Results are presented as mean±SD, p values were calculated by ordinary one-way ANOVA with Tukey correction for multiple comparisons.
