# Supplementary material for: Inhibition of HIV Replication by Apolipoprotein A-I Binding Protein Targeting the Lipid Rafts
Source: mBio. 2020 Jan 21;11(1):e02956-19. doi: 10.1128/mBio.02956-19 (PMC6974568; doi:10.1128/mBio.02956-19)
Supplement: FIG S3 [file mBio.02956-19-sf003.pdf]

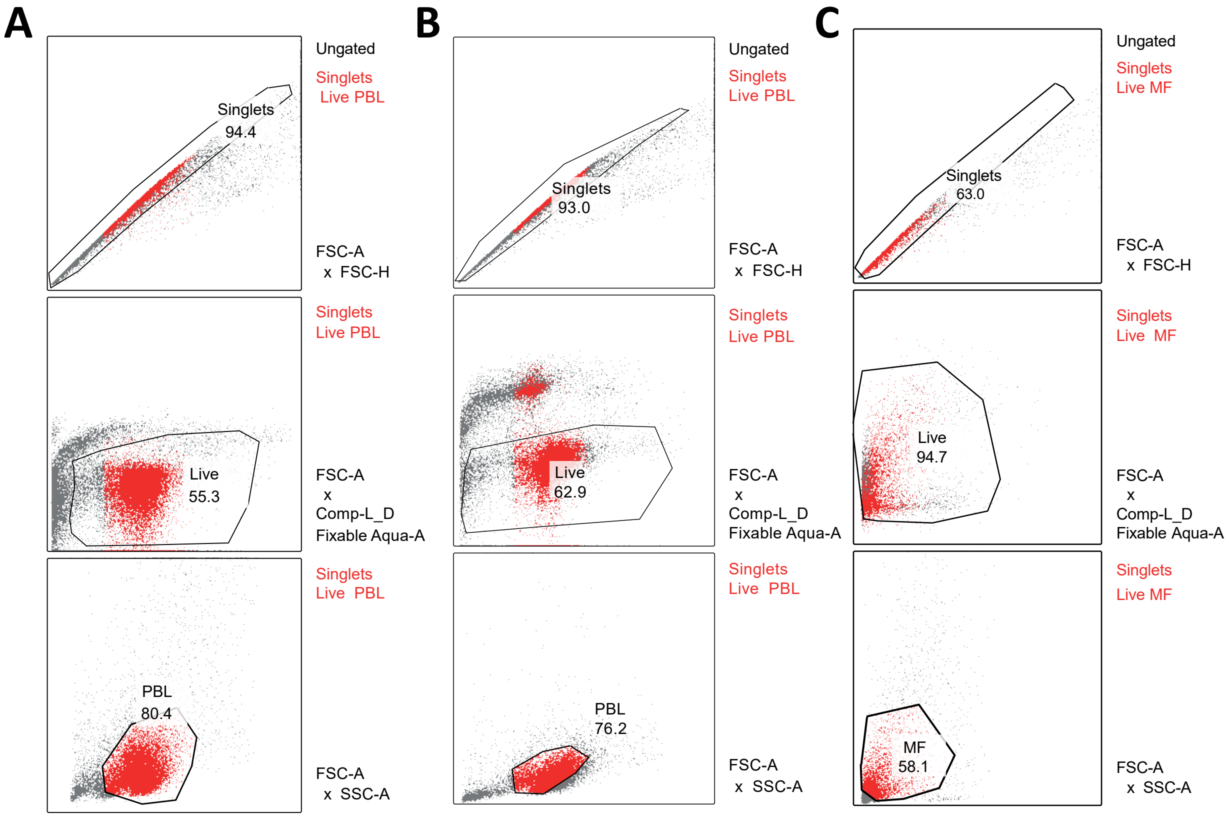

Figure S3. Gating strategy for Fig. 2. A – gating for unactivated PBL (Fig. 2A); B – gating for activated PBL (Fig. 2A)**;** C – gating for MDM (Fig. 2C).
