# Supplementary figures and images for: Inhibition of HIV Replication by Apolipoprotein A-I Binding Protein Targeting the Lipid Rafts
Source: mBio. 2020 Jan 21;11(1):e02956-19. doi: 10.1128/mBio.02956-19 (PMC6974568; doi:10.1128/mBio.02956-19)

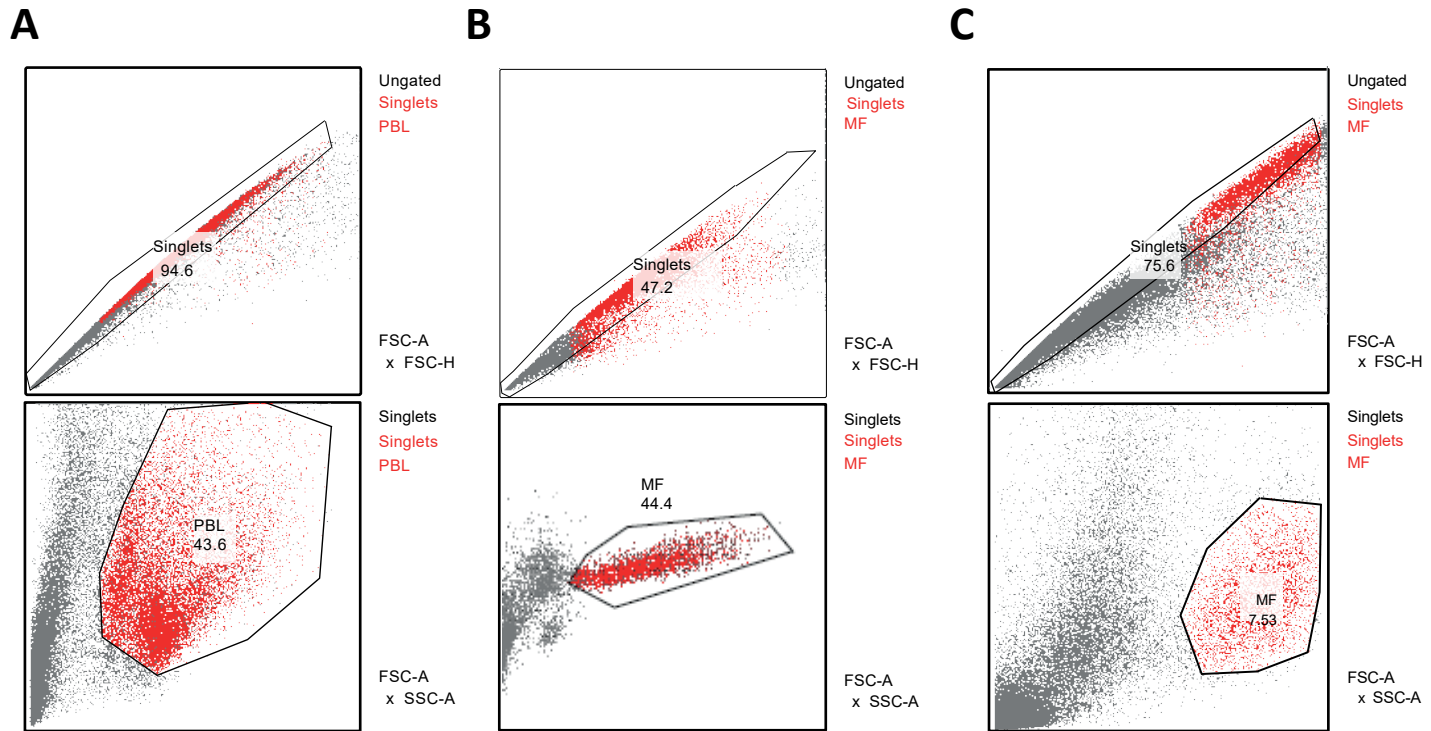

**Figure S4. Gating strategy for Fig. 3.** A – gating for PBL (Fig. 3A); B – gating for MDM (Fig. 3C); C – gating for MDM (Fig. 3F).

Supplement: FIG S4 [file mBio.02956-19-sf004.pdf]

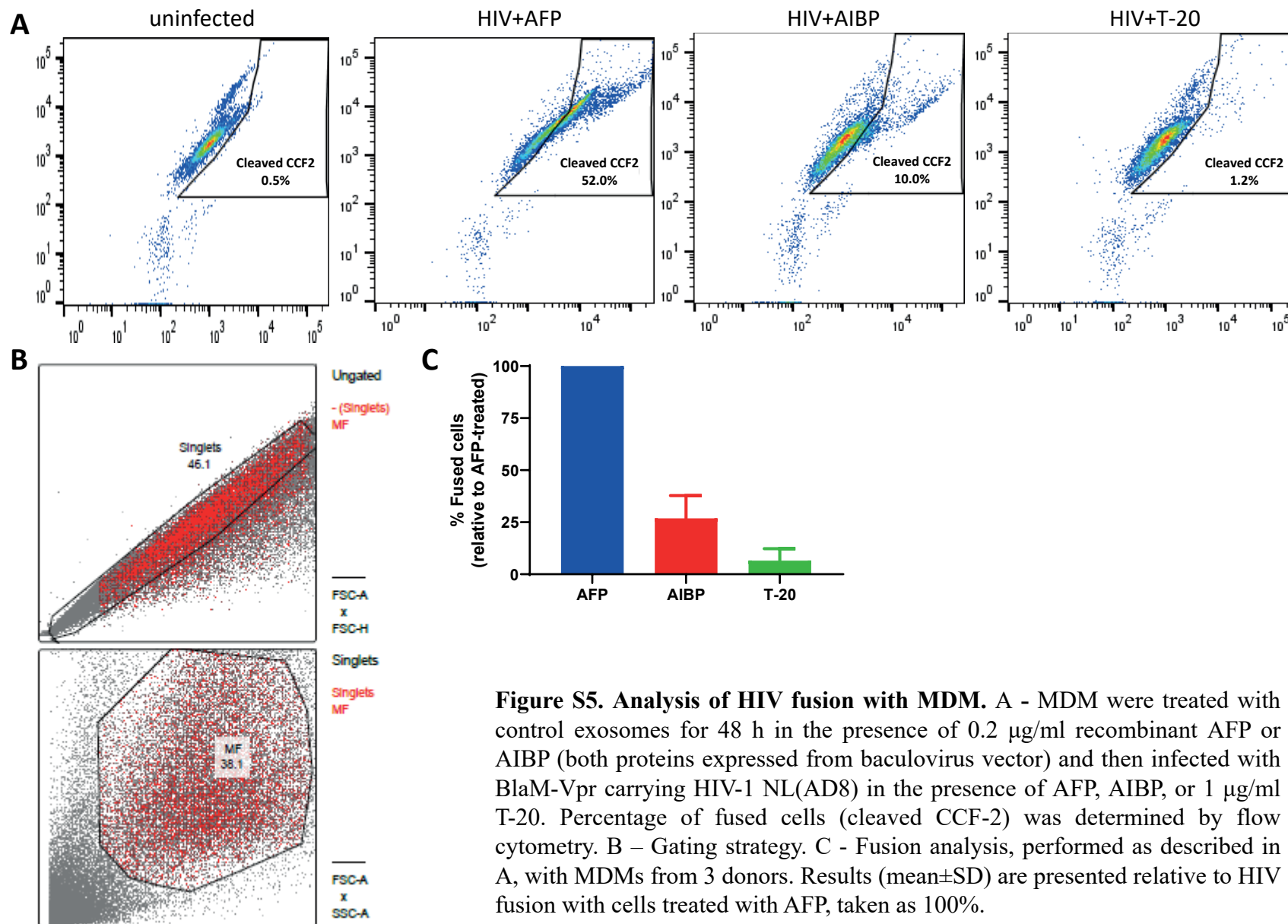

Supplement: FIG S5 [file mBio.02956-19-sf005.pdf]

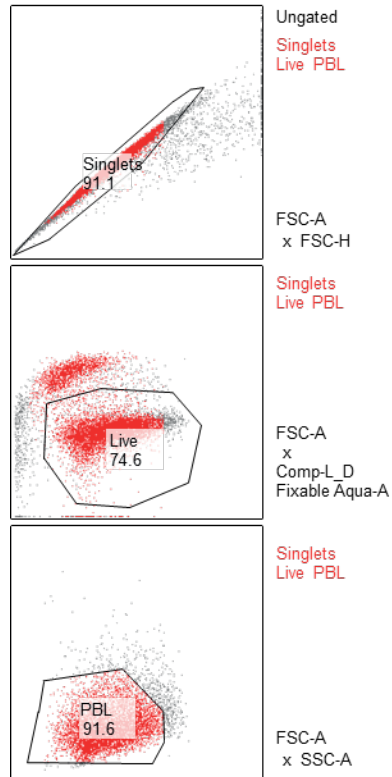

**Figure S6. Gating strategy for Fig. 5E.**

Supplement: FIG S6 [file mBio.02956-19-sf006.pdf]
