# Supplementary material for: Inhibition of HIV Replication by Apolipoprotein A-I Binding Protein Targeting the Lipid Rafts
Source: mBio. 2020 Jan 21;11(1):e02956-19. doi: 10.1128/mBio.02956-19 (PMC6974568; doi:10.1128/mBio.02956-19)
Supplement: FIG S1 [file mBio.02956-19-sf001.pdf]

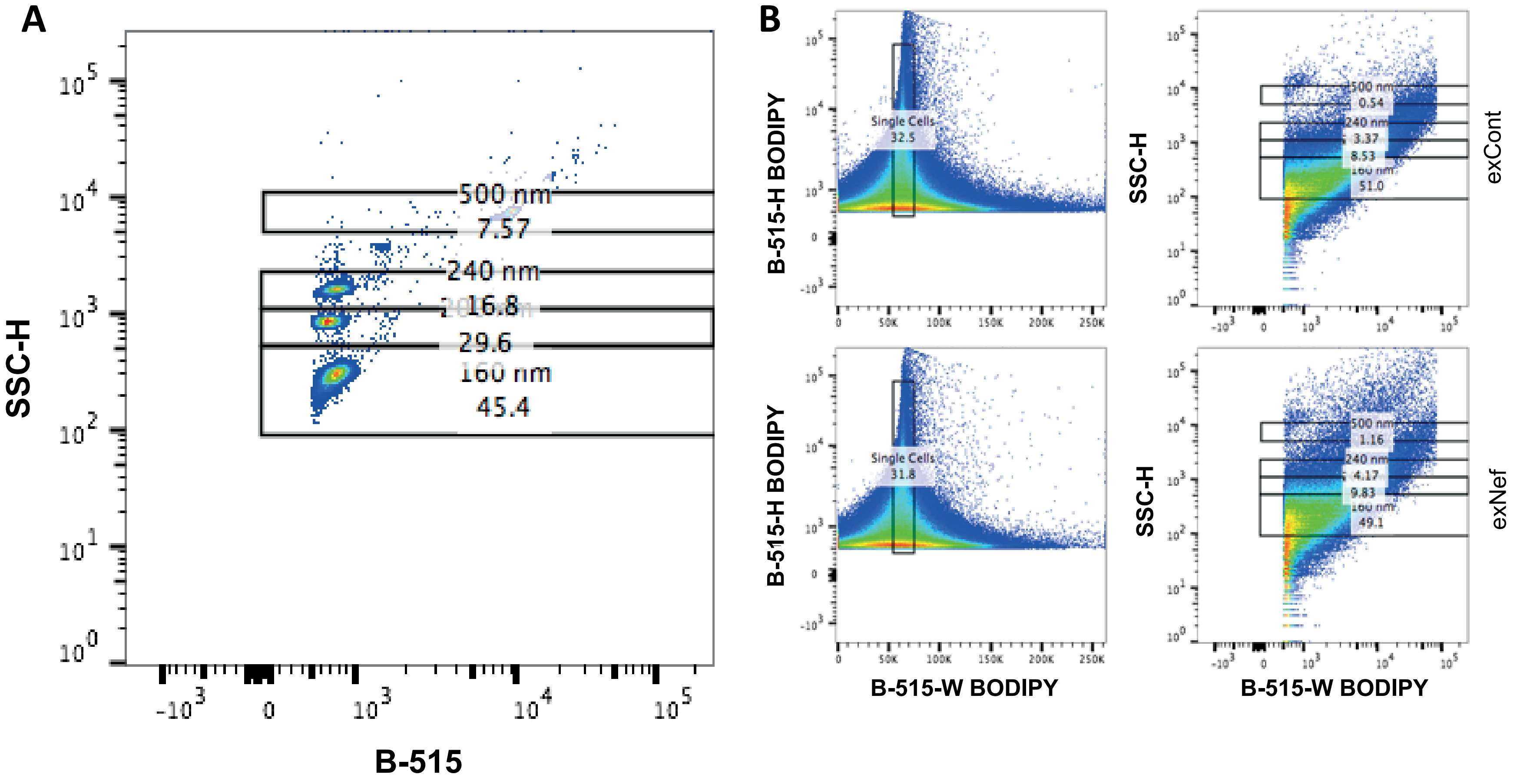

Figure S1: Visualization of EVs with flow cytometry. A **-** Defining sizing gates with Megamix beads. Fluorescent Megamix-plus SSC beads were used according to manufacturer (Cosmo Bio, CA). B - EV visualization with flow cytometry. exCont and exNef EVs were labeled with the lipophilic tracer BODIPY (Invitrogen, Life Technologies, CA) and visualized with a LSRII (Becton Dickinson) as BODIPY-positive events thresholding on BODIPY fluorescence. Left column: Gating strategy for flow analysis of BODIPY-labeled EVs isolated from mock- (upper panel) or Nef-transfected (lower panel) HEK293T cells. A singlet gate was defined by plotting fluorescence height versus fluorescence width. The gate excludes events with a high width and high height that represent aggregates. Right column: EV sizing as defined by Megamix-plus SSC beads gates (A). Results represent one of two similar experiments. On each plot, the fraction of total events in their respective gates is shown.
